# Adrenocorticotropic Hormone Therapy Improved Spasms and Sleep Disturbance in Smith–Magenis Syndrome: A Case Report

**DOI:** 10.3390/pediatric12030018

**Published:** 2020-10-24

**Authors:** Ken Momosaki, Jun Kido, Shirou Matsumoto, Shiro Ozasa, Kimitoshi Nakamura

**Affiliations:** Department of Pediatrics, Graduate School of Medical Sciences, Kumamoto University, Kumamoto 860-8556, Japan; momosaki@kuh.kumamoto-u.ac.jp (K.M.); s-pediat@gpo.kumamoto-u.ac.jp (S.M.); ozasas@kumamoto-u.ac.jp (S.O.); nakamura@kumamoot-u.ac.jp (K.N.)

**Keywords:** adrenocorticotropic hormone, corticotropin-releasing hormone, infantile spasms, sleep disturbance, Smith–Magenis syndrome

## Abstract

Smith–Magenis syndrome (SMS) is a complex disorder characterized by variable mental retardation, sleep disturbances, craniofacial and skeletal anomalies, self-injurious and attention-seeking behaviors, and speech and motor delays. The case of a 14-month-old girl with SMS who was experiencing spasm clusters and sleep disturbances with sleep–wake intervals of 1.5 to 2 h persisting from the neonatal period was examined. The patient’s spasms stopped and interictal electroencephalography did not show epileptic discharges after undergoing a high-dose adrenocorticotropic hormone (ACTH) therapy. Moreover, the patient’s sleep cycle stabilized 1 month after receiving the ACTH therapy. Dramatic reductions in the patient’s self-injurious behaviors were also noted. At 1 year following ACTH treatment, the patient’s improved sleep was maintained. High-dose ACTH treatment was considered to contribute to the normal adaptation of the hypothalamic–pituitary–adrenal axis by regulating the release of corticotropin-releasing hormone, resulting in improvement of the patient’s infantile spasms and sleep disturbances.

## 1. Introduction

Smith–Magenis syndrome (SMS) is a complex disorder characterized by variable mental retardation, sleep disturbances, craniofacial and skeletal anomalies, self-injurious and attention-seeking behaviors, and speech and motor delays [1]. SMS is associated with an interstitial deletion of chromosomal region 17p11.2, which contains the retinoic acid-induced 1 (*RAI1*) gene, or a mutation in the *RAI1* gene [1]. The incidence of SMS is estimated to be one per 15,000–25,000 births, though SMS is often underdiagnosed [2]. Epileptic seizures occur in 11–30% of individuals with SMS. [3,4] Moreover, 25% of patients with SMS reportedly present with electroencephalographic abnormalities, even in those without a clinical history of seizures [5]. However, patients with SMS who also present with infantile spasms are rare, and only a few such cases have been reported [6]. Recently, we experienced a patient with SMS who demonstrated improvements in the infantile spasms and sleep disturbance symptoms, after receiving a high dose of adrenocorticotropic hormone (ACTH) therapy. Here, we report the clinical course of this patient and discuss the mechanisms underlying the observed beneficial effects of ACTH therapy for infantile spasms and sleep disturbances.

## 2. Case Report

A female patient presented with infantile spasms characterized by forward-bending head (age, 14 months; height, 79.5 cm; weight, 11.3 kg; head circumference, 47.5 cm). The patient first presented with a cluster of spasms; however, within 1 month, the spasms gradually increased to 5–6 clusters per day. The patient presented with sleep disturbances, including a sleep–wake cycle of 1.5- to 2-h intervals, which persisted from the neonatal period, regardless of the time of day. At the age of 15 months, the patient was admitted to our institution owing to spasms and distinctive facial features.

The patient was able to maintain a sitting position at the age of 11 months and pull up to standing at the age of 15 months. Moreover, she had distinctive facial features with midface hypoplasia, upslanting palpebral fissures, frontal bossing, hypertelorism, cupid’s bow upper lip, synophrys, and low-set ears, and presented with brachydactyly and nail yanking lesions on both hands. The patient often displayed explosive anger and nail biting. G-banded cytogenetic analysis demonstrated an interstitial deletion of chromosome l7p11.2. The patient’s video electroencephalography (EEG) recordings obtained during a cluster of infantile spasms demonstrated diffuse slow waves and voltage attenuation. The interictal EEG did not reveal any type of hypsarrhythmia. Brain magnetic resonance imaging revealed mild enlargement in both lateral ventricles. 

**Figure 1:**
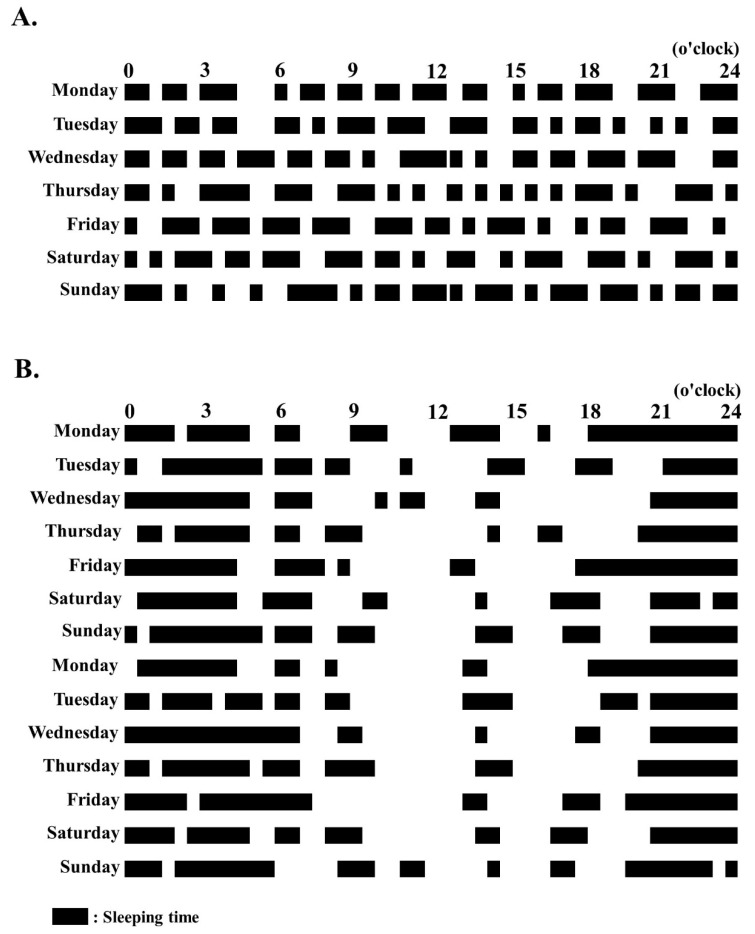
Sleep cycle in our patient with Smith–Magenis syndrome before and after adrenocorticotropic hormone (ACTH) treatment. (**A**) Before ACTH treatment. (**B**) At 6 months after ACTH treatment.

The patient was diagnosed with infantile spasms without hypsarrhythmia in SMS. The patient’s developmental quotients (DQs) on the Kyoto Scale of Psychological Development at the age of 16 months corresponded with those usually found at the age of 8 months in healthy controls (Postural–Motor DQ, 51; Cognitive–Adaptive DQ, 56; and Language–Social, 60). The patient initially received levetiracetam (20 mg/kg/day); however, the medication did not suppress the spasms. A high dose of ACTH (0.0125 mg/kg/day) was administered every day in the first 2 weeks, every 2 days in the forthcoming 2 weeks, two times for 1 week, and one time in the final week. Following ACTH therapy, the spasms stopped and no epileptic discharges were observed on the patient’s interictal EEG. Her sleep cycle stabilized at 1 month after receiving the ACTH therapy for 2 weeks, and the patient slept 5 to 6 h a night (Figure 1). The patient’s self-injurious behaviors were also dramatically reduced. At 1 year following ACTH treatment, the patient’s sleep improved and the spasms were controlled under receiving only levetiracetam treatment. 

## 3. Discussion

Our patient with SMS-developing infantile spasms, who presented with epileptic spasms and sleep disturbances, demonstrated improvement after receiving ACTH therapy. Infantile spasms are usually presented in children aged < 1 year and characterized by clusters or single epileptic spasms irrespective of with or without hypsarrhythmia, though infantile spasms without hypsarrhythmia are not common [7,8]. Deletion of *RAI1* is the primary contributor to the phenotype of SMS, which is characterized by circadian rhythm and sleep disturbances [9]. Furthermore, infantile spasms manifest as insults that enhance the intrinsic responses of the developing brain to stress. These responses are associated with the activated production and secretion of corticotropin-releasing hormone (CRH), a highly excitatory peptide [10]. High-dose ACTH treatment reduces CRH expression in different brain regions through the activation of steroid release, leading to negative feedback, and has direct actions on neurons through the activation of melanocortin receptors [11].

An unbalanced sleep–wake cycle has adverse effects on the stress system. Stress affects the relationship between sleep and metabolism through hypothalamic–pituitary–adrenal (HPA) axis activation. Sleep disturbances are also associated with malfunctioning changes in the HPA axis, leading to neuroendocrine dysregulation [12]. Alterations to the HPA axis may play a causative role in sleep disturbances, which may influence seizure control. Thus, effective treatment of sleep disturbances may improve seizure control [13].

However, the effect of a high-dose ACTH treatment could be affected by the duration of administration, patient’s age, and severity of spasm. Hino-Fukuyo et al [6] reported that 4 days of ACTH treatment in a patient resulted in improved infantile spasms. However, the patient eventually developed seizure, which was not controlled despite receiving a variety of antiepileptic drugs, and sleep disorder characterized by fragmented and shortened total sleep cycle.

Vigabatrin (VGB) is an effective drug for infantile spasm, which has been used in Europe since 1990s. It is considered to be the first choice for infantile spasm in nodular sclerosis [14]. VGB contributes to the brain gamma-aminobutyric acid (GABA) concentration increase by specifically and irreversibly inhibiting GABA transaminase, and has an anti-spasm effect. Moreover, Raol and Meti reported that VGB had a somnolence-inducing effect and might mediate its anticonvulsant effect by altering sleep architecture through sleep regulating areas [15]. In Japan, this drug was approved by the Ministry of Health, Labour, and Welfare in 2016. We could not administer VGB to this patient, as we did not obtain the approval to provide VGB to our patient. This drug may be effective for sleep disturbance in patients with SMS, as the spasms are controlled after receiving this medication. In the future, cumulative evidence from further cases is needed to evaluate the effects of ACTH and VGB treatments for infantile spasms and sleep disturbance in patients with SMS.

## 4. Conclusions

High-dose ACTH treatment was considered to contribute to normal adaptation of the HPA axis by regulation of CRH release, resulting in improvement in infantile spasms and sleep disturbance.

## 5. Statement of Ethics

The G-band cytogenetic analysis was performed in accordance with the Declaration of Helsinki and was approved by the Ethics Committee of our institution. Written informed consent was obtained from her parents.
